# Identification of Colon Cancer-Related RNAs Based on Heterogeneous Networks and Random Walk

**DOI:** 10.3390/biology11071003

**Published:** 2022-07-02

**Authors:** Bolin Chen, Teng Wang, Jinlei Zhang, Shengli Zhang, Xuequn Shang

**Affiliations:** 1 School of Computer Science, Northwestern Polytechnical University, Xi’an 710072, China; blchen@nwpu.edu.cn (B.C.); tengwang@mail.nwpu.edu.cn (T.W.); jinleizhang@mail.nwpu.edu.cn (J.Z.); 2 School of Information Technology, Minzu Normal University of Xingyi, Xingyi 562400, China; zhangshengli@xynun.edu.cn

**Keywords:** colon cancer, heterogeneous network, random walk, differential expression analysis

## Abstract

**Simple Summary:**

Colon cancer is a complex disease with high incidence rates and mortality worldwide. Although some medical methods have been used for screening, prevention and treatment, its molecular mechanism is still unclear. Among all dysfunctional factors, the change of mutual regulation relationship between RNAs is an important factor affecting the development of cancer. Therefore, the purpose of this study is to find RNAs related to colon cancer that have not been verified. We used differential expression analysis to screen mRNAs, miRNAs and lncRNAs and further constructed a heterogeneous interaction network among these three kinds of RNAs. The network propagation algorithm RW-DIR was then developed to mine the biological information contained in the network and to identify RNAs closely related to colon cancer. The research results have provided some theoretical support for disease research and provide a basis for narrowing the research scope of medical experiments.

**Abstract:**

Colon cancer is considered as a complex disease that consists of metastatic seeding in early stages. Such disease is not simply caused by the action of a single RNA, but is associated with disorders of many kinds of RNAs and their regulation relationships. Hence, it is of great significance to study the complex regulatory roles among mRNAs, miRNAs and lncRNAs for further understanding the pathogenic mechanism of colon cancer. In this study, we constructed a heterogeneous network consisting of differentially expressed mRNAs, miRNAs and lncRNAs. This contains three kinds of vertices and six types of edges. All RNAs were re-divided into three categories, which were “related”, “irrelevant” and “unlabeled”. They were processed by dynamic excitation restart random walk (RW-DIR) for identifying colon cancer-related RNAs. Ten RNAs were finally obtained related to colon cancer, which were hsa-miR-2682-5p, hsa-miR-1277-3p, ANGPTL1, SLC22A18AS, FENDRR, PHLPP2, hsa-miR-302a-5p, APCDD1, MEX3A and hsa-miR-509-3-5p. Numerical experiments have indicated that the proposed network construction framework and the following RW-DIR algorithm are effective for identifying colon cancer-related RNAs, and this kind of analysis framework can also be easily extended to other diseases, effectively narrowing the scope of biological experimental research.

## 1. Introduction

Due to the continuous improvement of medical standards, people’s life expectancy has increased. Currently, people’s diet has changed greatly and not only leads to higher incidences of cancer but also to younger individuals with higher incidence. Colon cancer is a common digestive tract malignant tumor occurring in the colon and about a tenth of all cancer cases, thus making it among the top three cancers in terms of incidence as well as mortality [1]. Metastatic seeding of colon cancer often occurs early when carcinoma is clinically undetectable and occurs years before diagnosis and surgery [2]. Although there are many effective screening means, a further understanding of its occurrence mechanism will promote the further development of innovative screening methods, prognostic indicators and treatment. However, the molecular mechanism of colon cancer formation is still not completely and clearly elucidated.

There are many discussions about the relationship among mRNAs, miRNAs, lncRNAs and diseases, because more and more studies show that these RNAs play key roles in many important biological processes and diseases. The association of mRNAs with cancers has been widely studied, and evidence has been accumulated, with the exception of the relationship of mRNAs and other non-coding RNAs. The microRNAs (miRNAs) are a class of non-coding small RNA molecules encoded by endogenous genes with about 22 nucleotides in length. In animals and plants, it is mainly involved in the regulation of post-transcriptional gene expression [3]. Benefiting from the regulatory function of miRNAs, there are many studies using miRNAs in building networks for identifying disease-related miRNAs, such as the BNPMDA algorithm [4] and NTSMDA algorithm [5]. Long non-coding RNAs (lncRNAs) are defined as RNAs that are longer than 200 nucleotides and that are not translated into functional proteins. It has been found that lncRNAs are closely related to cell cycle, such as differentiation, development, reproduction, aging and many human diseases [6,7]. With the increasing understanding and attention to lncRNAs, the use of network modelling to predict their relationship with diseases has also increased in recent years, such as the GANLDA algorithm [8] and the BPLLDA algorithm [9].

At present, increasing attention has been paid to the data mining algorithms of graphs. Among them, random walk is a very classic algorithm for mining graph structures, which has widely been used. Random walk(RW) models have also been applied in various domains, such as locomotion and the foraging of animals, the dynamics of neuronal firing and decision-making in the brain, descriptions of financial markets, evolution of research interests ranking systems, dimension reduction and feature extraction from high-dimensional data and even sports statistics. RW theory can also help predict the arrival times of diseases spreading in networks [10].

Many current methods for analyzing RNA interaction networks ignore the heterogeneous characteristics of the network. They either only use the interactions between two types of RNAs, which ignore the interactions within the same type of RNA [11], or do not treat different types of RNAs (nodes) differently, which render the obtained results in a state of non-equilibrium. For instance, the label reasoning models often need to calculate entropy, but they cannot conduct the global random at the same time [12]. To overcome of this, this study proposes to combine the idea of maximum entropy with a tag inference by using random walk to identify key RNAs related to colon cancer by considering the overall property of mRNAs, miRNAs, and lncRNAs in the heterogeneous network. The results of different types of RNAs were balanced.

**Figure 1:**
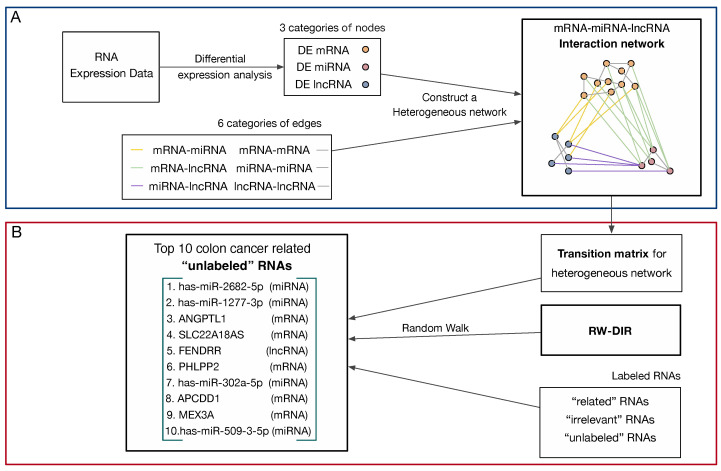
This is the workflow of this study. (**A**) Construct the mRNA-miRNA-lncRNA interaction network. (**B**) RW-DIR algorithm was applied to obtain the results of colon cancer-related RNAs.

To be more specific, this study first proposed to construct a colon cancer-specific RNA interaction heterogeneous network. The traditional random walk algorithm was then improved to find and analyze the RNAs related to colon cancer. The details are provided as follows. Firstly, we constructed a heterogeneous biological network for colon cancer, in which mRNAs, miRNAs and lncRNAs are the vertices of the network, and the interactions between every two types of RNAs and within each RNA served as the edges. There were three types of vertices and six kinds of edges. Then, we designed a random walk transfer matrix for heterogeneous networks, and labelled all vertices as three categories, namely “related”, “irrelevant” and “unlabeled”, according to whether the vertices are related to colon cancer. Applying the idea of traditional random walk, different measures were taken for different category vertices encountered in the process of walking so as to achieve the purpose of classifying the “unlabeled” RNAs. Figure 1 indicates the processes of identifying colon cancer-related RNAs in this study, where part A is the process of building the heterogeneous network, and part B is the process of RW-DIR.

## 2. Materials and Methods

### 2.1. RNA Expression Data

In this study, mRNA expression data, miRNA expression data, lncRNA expression data and clinical data were collected from the open-access dataset of The Cancer Genome Atlas (TCGA) database [13]. The project ID was “TCGA-COAD”.

For mRNA and lncRNA expression data, the experimental strategy we downloaded was “RNA-Seq”, the data type was “Gene Expression Quantification”, and the data category was “transcriptome profiling”. The data from the Ensemble database [14] annotated the type of all RNAs in the gene expression data, and it was downloaded from TCGA. In this study, we selected “protein-coding gene” and “lncRNA” as mRNA and lncRNA for subsequent analysis.

For miRNA, the data type we downloaded was “Isoform Expression Quantification”, the workflow type was “BCGSC miRNA Profiling”, and the data category was “Transcriptome Profiling”.

The clinical data of colon cancer were also obtained from TCGA. The original clinical data contained a variety of clinical information items of the samples, and only the information about sample ID and cancer stage was selected. The sample ID was used to map the RNA expression data of the particular sample, and the information of the cancer stage was used to distinguish whether the samples were cancerous or paracancerous tissue; the latter will be used as normal samples.

### 2.2. The Relationship of RNAs

The connection in this study could divide into two categories. One was the connection between different kinds of RNA, and the other was the connection within the same type of RNA. The relationship and data source databases are shown in Table 1. The relationships between “miRNA-miRNA” and “lncRNA-lncRNA” are obtained by the Deepwalk algorithm [15], and their respective associations are related to their target genes.

### 2.3. Classification of RNA

The mRNAs, miRNAs, and lncRNAs that are related to colon cancer and are verified by experiments were obtained from the databases shown in Table 2. We selected known colon cancer-related RNAs as RNAs with “related” labels and randomly selected an equal amount of RNAs that related to other diseases and excluded colon cancer-related RNAs as RNAs with an “irrelevant” label. The remaining vertices were marked with the “unlabeled” label.

### 2.4. Data Preprocessing

Since deeper sequencing always produces more sequence fragments, in differential expression analysis, the row counts were rarely used directly. In practice, the counts are usually normalized to eliminate sequencing differences due to sequencing depth. The log-CPM normalization method was used in this study.

The R package edgeR [23] was used for data preprocessing. In all datasets, there would be a mixture of expressed genes and non-expressed genes. Reducing these noises would not only significantly improve the accuracy of statistical inferences from RNA-seq but also allow mathematical models in the data to be more accurately estimated and reduce the amount of RNA analyzed downstream; thus, this study used the “filterByExpr” function in edgeR package to filter RNAs with low expression counts [24]. For each group of data, the TMM (Trimmed Mean of M-values) [25] algorithm was also considered to ensure that each sample has a similar distribution of expression data. After the data preprocessing, the number of three RNAs and the number of samples in their respective datasets are shown in Table 3.

### 2.5. Differential Expression Analysis

Differential expression analysis [23] refers to obtaining normalized read count data and performing statistical analysis to discover quantitative changes in expression levels between experimental groups. There were two main parameters for using this method to screen differentially expressed RNAs: one is |logFC| and the other one is the *p*-value.

In this study, the exact test method that is based on the negative binomial distribution in R package. EdgeR was used to identify differentially expressed RNAs. The threshold selection of the three differentially expressed RNAs was different. Specifically, the mRNA that had *p*-value < 0.05 and |logFC|≥2 could be chosen as the differentially expressed (DE) mRNA; the miRNA that had *p*-value < 0.05 and |logFC|≥2 could be chosen as the DE miRNA; the lncRNA that had *p*-value < 0.05 and |logFC|≥1 could be chosen as the DE lncRNA. Finally, 1372 DE mRNAs, 175 DE miRNAs and 137 DE lncRNAs were obtained in this study.

### 2.6. Construct Heterogeneous Network

**Figure 2:**
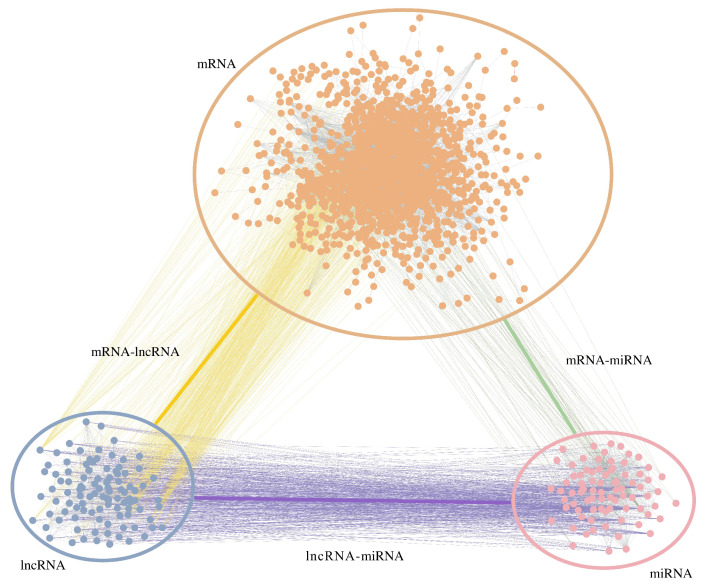
The heterogeneous network of RNA interactions. Orange vertices are mRNAs, pink vertices are miRNAs, and blue vertices are lncRNAs. The grey edges represent the connection within the same kind of RNAs, the yellow edges represent the relationship between mRNA and lncRNA, the green edges represent the relationship between mRNA and miRNA, and the purple edges represent the relationship between miRNA and lncRNA.

This study was based on the exploration of the complex regulatory relationship among mRNAs, miRNAs and lncRNAs. Therefore, a heterogeneous network was first constructed to express the relationship among the three in the form of a network for subsequent data mining. The heterogeneous network was shown in Figure 2. In this study, this network was defined as G(V,E), where V represents all vertices and E represents all edges. The adjacency matrix H can be obtained by assigning a value of 1 if there was an edge between two vertices, and it is 0 otherwise.

### 2.7. RW-DIR

**Figure 3:**
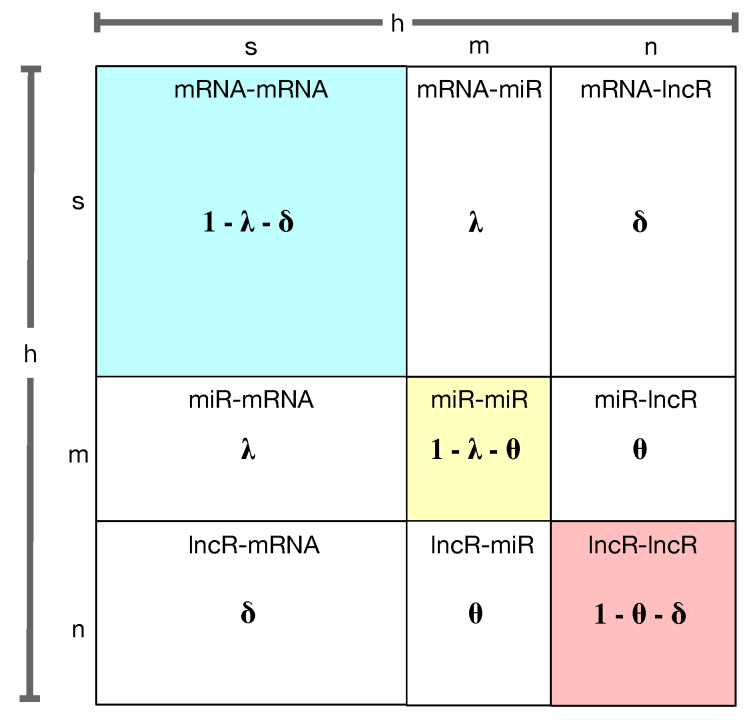
This is a diagram of the transition matrix calculation. Nine sub-matrices form a complete transition matrix. λ is the transfer parameter between mRNA and miRNA, δ is the transfer parameter between mRNA and lncRNA, and θ is the transfer parameter between miRNA and lncRNA. The transfer parameters in each small square correspond to the parameters that could be used to calculate the percentage of summarized weight corresponding to the sub-transition matrix.

Similarly to traditional random walk, RW-DIR required a transition matrix [26] for the subsequent walk in the network, but the transition matrix in this study was designed based on heterogeneous networks. A diagram of the calculation method is shown in Figure 3. In the figure, *s* represents the number of mRNAs, *m* represents the number of miRNAs, *n* represents the number of lncRNAs, and  *h* represents the number of all RNAs. Obviously, *h* equals to the sum of *s*, *m*, and *n*. Three parameters, λ, δ and θ, are used to adjust the transition probability of different types of RNAs. The λ is the transfer parameter between mRNA and miRNA, the δ is the transfer parameter between mRNA and lncRNA, and  the θ is the transfer parameter between miRNA and lncRNA. Specifically, we have the following.

(1) λ=|mRNA−miRNA|/(|mRNA−mRNA|+|miRNA−miRNA|)

(2) δ=|mRNA−lncRNA|/(|mRNA−mRNA|+|lncRNA−lncRNA|)

(3) θ=|miRNA−lncRNA|/(|miRNA−miRNA|+|lncRNA−lncRNA|)

The method to calculate transition matrix W is written as Equation (4):

(4) $$W\left(i,j\right)=X\cdot \frac{H(i,j)}{\sum_{k=a}^{b}H\left(i,k\right)},$$

where X={x∣x∈{A,(1−A−B)},A≠B}}, {A,B}∈{λ,δ,θ}, {i,j}∈{{1,…,s},{s+1,…,s+m},{s+m+1,…,h}}, a∈{1,s+1,s+m+1} and b∈{s,s+m,h}. The selection of parameter *X*, *a* and *b* could be more intuitive according to the Figure 3. Since H was comprised 9 sub-matrices, the sub-matrices should be calculated separately.

Random walk is a discrete-time Markov process where a walker located at vertex *i* at a certain moment will jump to adjacent vertex *j* at the next moment with probability W(i,j). This jump is independent to the past. Before exhibiting random walk with dynamic incentive restart (RW-DIR), we introduced the random walk with restart (RWR) method [27] first. The difference between RWR and traditional random walk is that there is a certain probability of returning to the starting point after each step of walking. In RWR, based on the transition matrix *W* and the hopping process P(t+1) = αWP(t)+(1−α)P(0), where P(t) is a vector that represents the probability of walker at all vertex at time *t*, and P(t) will converge, which means RWR will reach a stationary distribution.

Next, we would introduce RW-DIR. The vertices in the studied heterogeneous network have certain prior knowledge about colon cancer; thus, we considered that in the process of walking. Different measures should be taken for vertices with different labels such that the reliable colon cancer-related vertices can be finally obtained. The specific algorithm process is defined in Algorithm 1. In the initial round of the algorithm, we assigned the value of 1 in $P_{0}$ to the colon cancer-related RNA with the largest degree, and  the rest was 0. In each round of the algorithm, judging which vertices the current round walks to was based on whether there were any changes in P(t) compared with P(t−1). Considering that the random walk process could spread the information of labelled RNAs, we used known knowledge for random walk and information dissemination. We inflated the effect of the “related” vertex, which was gradually added to $P_{0}$. It enhanced its global influence in the process of repeatedly restarting. We shrunk the effect of the “irrelevant” vertex, which included adding a penalty value to the “irrelevant” vertex in each iteration to make it smaller; this would reduce their impact of the global process. For the “unlabeled” vertex, we referred to the idea of maximum entropy by calculating the entropy value of each vertex after each iteration, and the inference result of the vertex with the largest entropy value represented the highest uncertainty. The transition probability matrix *W* is recalculated according to the entropy value in each round iteration. The larger the entropy value, the larger transition probability matrix value of the vertex. It should be noted that the calculation of entropy needs to be started after walking to the global process; that is, the calculation of the transfer matrix also needs to wait until the process of walking to all vertices. It would help the vertex with the “unlabeled” result in collecting more information for further inferences.
**Algorithm 1** Random Walk with Dynamic Incentive Restart (RW-DIR)
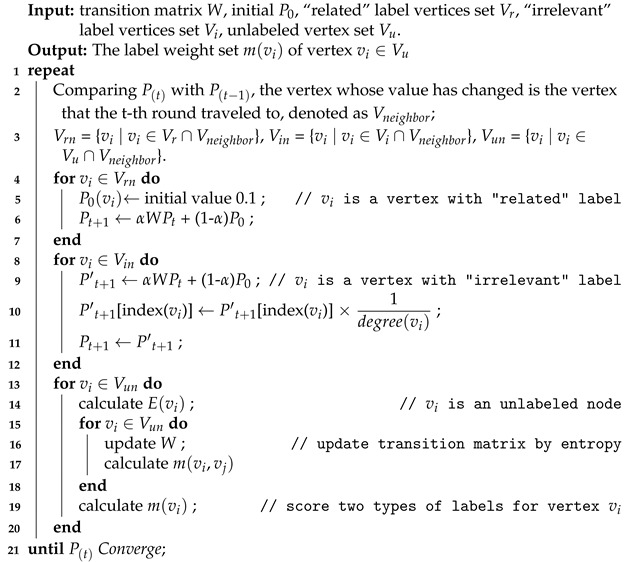


The entropy value $E(v_{j})$ of the vertex $v_{j}$ can be calculated by Equation (5), where $m_{k}\left(v_{i}\right)$ represents the possibility that vertex $v_{i}$ belonged to label *k*. In this study, we set k=1 represent the “related” vertex, and k=2 represent the “irrelevant” vertex. When the $E(v_{i})$ value of vertex $v_{i}$ is calculated for the first time (the *t*th round walk), where $v_{i}$ was the “unlabeled” vertex, we obtain initial $m_{1}\left(v_{i}\right)$ = $\frac{h-ascending\;rank\;of\;P_{t}\left[index\left(v_{i}\right)\right]}{h}$, and $m_{2}\left(v_{i}\right)$ = 1−$m_{1}\left(v_{i}\right)$. For “related” vertex $v_{r}$, we set $m_{1}\left(v_{r}\right)$ = 1 and $m_{2}\left(v_{r}\right)$ = 0. For “irrelevant” vertex $v_{l}$, we set $m_{2}\left(v_{l}\right)$ = 1 and $m_{1}\left(v_{l}\right)$ = 0.

(5) $$E\left(v_{i}\right)=-\sum_{k=1}^{2}m_{k}\left(v_{i}\right)log_{2}m_{k}\left(v_{i}\right),$$

When the random walk process had covered the entire network (in the *t*th round walk), transition matrix *W* needed to be updated according to the entropy value of E by Equation (6), where $v_{j}$ represented all neighbor vertices of $v_{i}$, including $v_{i}$.

(6) $$W\left(v_{i},v_{j}\right)=\frac{E(v_{i})}{\sum_{v_{j}\in N^{+}\left(v_{i}\right)}E\left(v_{j}\right)},$$

After that, it was necessary to update and calculate the probability that each vertex belonged to each label. As shown in Equation (7), $m_{k}\left(v_{i},v_{j}\right)$ represented the probability that label k propagated from vertex $v_{i}$ to vertex $v_{j}$, which could be used to further calculate the probability of vertex $v_{i}$ with label k, i.e., $m_{k}\left(v_{i}\right)$, in Equation (8).

(7) $$m_{k}\left(v_{i},v_{j}\right)=m_{k}\left(v_{i}\right)\times W_{v_{i},v_{j}},$$

(8) $$m_{k}\left(v_{i}\right)=\frac{\sum_{v_{j}\in N^{-}\left(v_{i}\right)}m_{k}\left(v_{i},v_{j}\right)}{\sum_{k=1}^{2}\sum_{v_{j}\in N^{-}\left(v_{i}\right)}m_{k}\left(v_{i},v_{j}\right)},$$

In summary, at the beginning of the algorithm, we started the RW-DIR algorithm with one known colon cancer-related vertex. In the subsequent iterative process of the walker, compared to the previous round, we classified the type of vertices that had been “walked”. If it was a “related” vertex that has never been reached before, it would be added to P0 and assigned a value of 0.1. If it was an “irrelevant” label, add a penalty value to $P_{t}$; that is, we divide it by its degree. If the walker “walked” to vertices with no label, its entropy value would be calculated, and the transition matrix should be updated by the entropy value. Finally, the algorithm would stop after $P_{t}$ convergence. The $m_{1}$ sorting result of the unlabeled vertex will be used as an indicator for screening colon cancer-related RNAs.

## 3. Results

### 3.1. The Heterogeneous Network

The relationships of “miRNA-miRNA” and “lncRNA-lncRNA” were obtained by constructing the interaction network of their respective target genes and then applying the Deepwalk algorithm. After applied the Deepwalk algorithm in miRNA-target genes interaction networks, we obtained 273,431 edges and 740 miRNA vertices. Moreover, the miRNA-target gene database was miRtarbase. The DE miRNAs obtained in this study were screened out; finally, the miRNA functional similarity network had 81 vertices and 389 edges, which was shown in the pink circle part in Figure 2. We also applied the Deepwalk algorithm in the lncRNA-target genes interaction network. The lncRNA-target gene database was starBase, and the relationship of “lncRNA-target” was screened with a threshold greater than 0.5; we obtained 63,546 edges and 357 lncRNA vertices. In this study, we set the edge weight of the threshold value to be greater than 0.9 in the network and selected DE lncRNAs. Finally, we obtained 65 lncRNA vertices and 604 edges, which are shown in the blue circle in Figure 2.

The RNA interaction heterogeneous network constructed in this study had 1521 vertices and 9651 edges, as shown in Figure 2. Among them, the number of mRNA vertices was 1340, the number of miRNA vertices was 80, and the number of lncRNA vertices was 101. The edge’s information is shown in Table 4.

### 3.2. The Result of RW-DIR

We selected the top 10 RNAs in descending order with respect to the $m_{1}$ value as candidate colon cancer-related RNAs, where $m_{1}$ represented the probability that the vertex was classified as colon cancer-related RNAs. These RNAs were hsa-miR-2682-5p, hsa-miR-1277-3p, ANGPTL1, SLC22A18AS, FENDRR, PHLPP2, hsa-miR-302a-5p, APCDD1, MEX3A and hsa-miR-509-3-5p. Among them, FENDRR is lncRNA; hsa-miR-2682-5p, hsa-miR-1277-3p, hsa-miR-302a-5p and hsa-miR-509-3-5p are miRNAs; ANGPTL1, SLC22A18AS, PHLPP2, APCDD1 and MEX3A are mRNAs.

#### 3.2.1. Colon Cancer Related mRNAs

ANGPTL is a family of proteins similar to angiopoietins. They affect angiogenesis, inflammation, metabolic disturbances, hematopoiesis, and cancer development. Studies have shown that ANGPTL1 can act as an anti-angiogenic factor and a tumor suppressor [28]. ANGPTL1 has been reported to suppress migration and invasion in lung, breast and colorectal cancer, acting as a novel tumor suppressor candidate [29]. For SLC22A18AS, high expression levels are significantly associated with worsening disease progression. In addition, low levels of SLC22A18AS are also correlated with better overall survival for lung adenocarcinoma patients [30]. For PHLPP2, maintaining balanced PHLPP2 expression levels is critical for disease prevention, as changes in steady-state levels of PHLPP2 are associated with many diseases, including diabetes, hepatic steatosis, and cancer. Recently, many studies have shown that the expression of PHLPP2 is universally absent in a variety of cancers and plays a key role in a wide range of biological processes, including cancer cell proliferation, metastasis, autophagy and apoptosis [31]. For APCDD1, there is a study that suggested that APCDD1 regulated breast cancer progression by targeting canonical WNT signaling and modulating breast cancer cell invasion [32]. For MEX3A, it may promote glioma development by regulating cell proliferation, cell apoptosis, cell cycle and cell migration, and MEX3A has been identified as a potential tumor promoter in glioma development and therapeutic target in glioma treatment [33]. Taken together, these mRNAs are all related to the survival process of cells and play important roles in some cancers.

#### 3.2.2. Colon Cancer Related miRNAs

Hsa-miR-2682-5p and hsa-miR-1277-3p are the top two results. The neighbor vertices of hsa-miR-2682-5p and hsa-miR-1277-3p in the heterogeneous network, which were constructed in this study, were all “related” vertices. For hsa-miR-2682-5p, the study has suggested that miR-2682-5p promotes cell proliferation and migration in oral squamous cell carcinoma, and its target mRNA and lncRNA in this study were all known colon cancer-related RNAs [34]. For hsa-miR-302a-5p, some studies showed that the miR-302 family, which includes miR-302b, miR-302c, and miR-302d, exerts antitumor effects in several cancers, such as endometrial carcinoma, glioma and breast cancer. MiR-302a has been shown to function as a tumor suppressor by regulating diverse cellular functions [35]. For example, HMGA2 has been implicated as a driver of tumor metastasis; however, hsa-miR-302a-5p is the powerful post-transcriptional regulator of HMGA2 [36]. For hsa-miR-509-3-5p, the decreased expression of miR-509-3-5P promoted the colony, migration and invasion abilities of gastric cancer cells in vitro as well as tumorigenesis and lymph vertex metastasis in vivo [37]. In summary, the regulation of miRNAs on cancer is generally reflected in the regulation of their target genes. The four candidate colon cancer-related miRNAs obtained in this study were basically closely related to the occurrence of some common cancers, and their relationship with colon cancer deserves further study.

#### 3.2.3. Colon Cancer Related lncRNAs

Table 5 has summarized the top 10 RNAs and related diseases. We can see from the table that FENDRR is the only lncRNA among the top 10 candidate colon cancer-related RNAs. Studies have shown that the low expression of the FENDRR occurs in gastric cancer and is associated with poor prognosis; thus, FENDRR plays an important role in the progression and metastasis of gastric cancer [38]. FENDRR is expressed in a variety of cancers and is significantly associated with different clinical features. Furthermore, FENDRR has shown potential as a biomarker for cancer diagnosis, prognosis and treatment. Therefore, FENDRR is a potential candidate lncRNA for studying colon cancer-related RNAs [39].

### 3.3. Performance of RW-DIR

**Figure 4:**
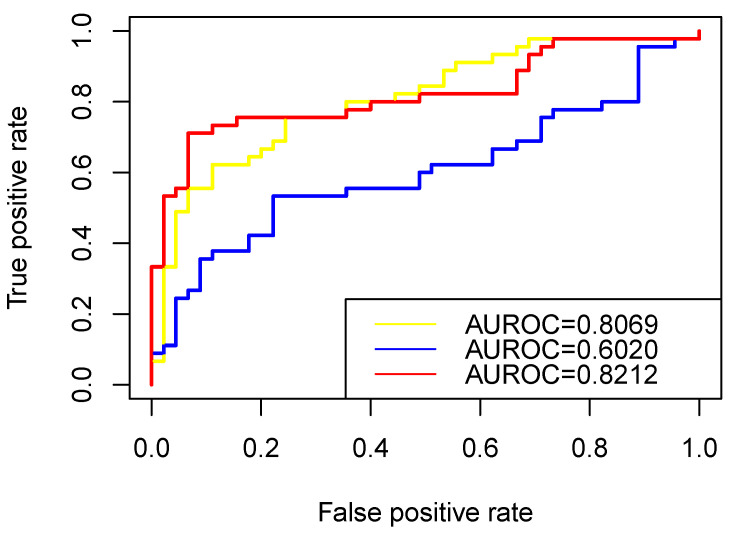
The prediction performance for prioritizing colon cancer causal RNAs. The ROC curves illustrating the performance in distinguishing “related” label RNAs from “irrelevant” label RNAs. Red curve represented RW-DIR; the blue curve represented TRWR, which was directly used on the basis of the heterogeneous network that is constructed in this study; the yellow curve represented RW-DIR without entropy.

In this study, the ROC curve was used to visually display the classification performance of the algorithm, and the AUC value was used to measure the classification ability of the algorithm [40]. In this study, we used LOO-CV(Leave-One-Out Cross-Validation) to test the performance of RW-DIR. Specifically, we placed one “related” vertex into “unlabeled” vertices each time and tested its $m_{1}$ value. Equal amounts of RNAs were randomly selected from the candidate RNAs related to other cancers, with the exception of colon cancer as negative samples, and checked their $m_{1}$ value. After the results were obtained, the ROC diagram was made, as shown in Figure 4, in which the color of the curve is red, and AUC is 0.8212.

We also evaluated the performance of the traditional restart random walk (TRWR) algorithm [27] on heterogeneous networks with a traditional transition matrix and the performance of the RW-DIR algorithm without using entropy. In detail, the process of using TRWR algorithm on the RNA interaction heterogeneous network was as follows: first, in order to obtained the relationship between “unlabeled” RNAs and “related” RNAs, the “related” vertices in P0 all were assigned a value of 0.1, and the transition matrix was calculated according to the degree of the vertex that needed to meet the standardization rules of the transition matrix; finally, walker can walk according to Equation (6) until convergence. The leave-one-out method was used during testing and the ROC diagram is shown in Figure 4, and the color of its curve is blue.

(9) $$P_{t+1}=\alpha WP_{t}+\left(1-\alpha \right)P_{0}.$$

The yellow curve in Figure 4 referred to the method that lacked the part of entropy in RW-DIR (lack of processing for “unlabeled” nodes). Specifically, the idea of not considering maximum entropy was to only consider RNAs that are known to be associated with colon cancer, and we only expand or shrink these vertices at this time, and perform nothing else for the “unlabeled” vertex. We also performed the leave-one-out to test the performance of the method.

It can be seen from the results that designing a transfer matrix for heterogeneous networks is very necessary for the network’s propagation algorithm, which can try to avoid the deviation of the results caused by the difference in the number of different types of vertices or edges. For the three types of RNAs, the number of mRNAs was far greater than that of miRNAs and lncRNAs, and the number of edges in different subnetworks was also very different. On the other hand, it was reasonable to use the idea of maximum entropy for final RNA screening. If only the walk probability ($P_{t}$) ranking was used as the final result, the aggregation or dispersion of vertices will be ignored, and the results were average, while in this study, the $m_{1}$ value was used as the parameter for the final comparison, which was obtained by aggregating the information of the global vertices and not the score that transferred from the rank of $P_{t}$. Comparing the AUC values of the three methods, it could be seen that RW-DIR performed the best, and RWR performed poorly.

## 4. Discussion

Colon cancer has become the third most common cancer in the world. In recent years, the characteristics of its younger patient population, urbanization and easy metastasis in the early stage have attracted our attention. At present, its molecular mechanism is still unclear. Cancer-related RNAs are the key to targeted therapy. Therefore, this study is committed to find mRNAs, miRNAs and lncRNAs that are closely related to colon cancer. Based on RNA expression data, we analyzed and mined it at the data level and topology level in order to obtain relevant results and applied them to medical experiments, provide data evidence and narrow the research scope of medical experiments.

In this study, we started with RNA expression data and conducted differential expression analysis to obtain DEmRNA, DEmiRNA and DElncRNA. Combined with the RNA interaction database and the graph-embedding method, the heterogeneous network of mRNA-miRNA-lncRNA interaction was constructed. On this basis, we designed an innovative network propagation and data mining algorithm. The main idea is to treat the vertices with different types and labels differently, and finally, we obtained the relevant RNAs that are most related to colon cancer but not confirmed by research. We obtained the top ten unproven RNAs associated with colon cancer. They are hsa-miR-2682-5p, hsa-miR-1277-3p, ANGPTL1, SLC22A18AS, FENDRR, PHLPP2, hsa-miR-302a-5p, APCDD1, MEX3A and hsa-miR-509-3-5p. Moreover, most of them have a certain inhibitory effect on the development of other types of cancer, and some can even be used as biomarkers.

For miRNAs in the results, there was increasing evidence that indicated that hsa-miR-2682-5p acted as a tumor suppressor in various cancers, such as non-small cell lung cancer (NSCLC) and Pancreatic cancer (PC) [41,42]; hsa-miR-302a-5p also suppresses proliferation and invasion in NSCLC [35], and hsa-miR-509-3-5p can suppress lung cancer by inhibiting the proliferation and migration of lung cancer cells [37]. The first two are regulated by targeting mRNA, while the last one regulates cancer cells through the relationship with lncRNA, which also showed that the competitive and cooperative relationship between different RNAs was close and further strengthens the possibility that the experimental results are likely to be related to colon cancer. For the mRNA results obtained in the study, we found that the mRNA of the top four has been experimentally verified, and when it is highly expressed in other types of cancer, it has positive significance for the development and prognosis of cancer. The top four mRNAs are ANGPTL1, SLC22A18AS, PHLPP2 and APCDD1. They have proved that they could inhibit the proliferation and metastasis of cancer cells in many other cancers, such as lung cancer, breast cancer and colon cancer. The last ranked mRNA, MEX3A [33], is a promoter for glioma and a therapeutic target in the treatment of glioma.

The model constructed in this study needs to be supported by a large number of databases. For some diseases, the amount of data may not be large enough, resulting in inaccurate results in data mining. However, at present, the cancer-related databases are relatively complete, and the information about RNA is relatively perfect. Therefore, most common cancers can find relevant mRNA, miRNA and lncRNA by this method. Moreover, the current RNA interaction network was built on the basis of differentially expressed RNA, and some cancer-related RNAs had not been screened by differential expression analysis. In addition, there are still some problems in using machine learning and other computing methods to identify cancer-related RNAs, such as little data quantity, unbalanced sample data, and difficult modeling. Moreover, it still requires follow-up biological experiments for further verification. Therefore, it is necessary to find a better method to screen the vertices of the interaction network in the future.

The network topology model and global heterogeneous network analysis algorithm proposed in this study provided new inspiration and ideas for finding RNA related to colon cancer and other diseases. Although there were some limitations in the data, they still did not affect the reliability of the final result.

## 5. Conclusions

Colon cancer is a complex disease with a high incidence rate and high mortality. Although there are certain medical methods for its prevention and treatment, its molecular mechanism has not been clear. The complex regulation between RNAs is an important cause of cancer. Therefore, the purpose of this study is to find RNA related to colon cancer that has not been verified. We used the regulatory relationship between mRNA, miRNA and lncRNA screened by differential expression analysis to construct a heterogeneous network, and then we analyzed its topological characteristics and used the RW-DIR method to find RNAs that are closely related to colon cancer. The results can provide some theoretical support for disease research and provide a basis for medical experiments.

## Figures and Tables

**Table 1:** RNA association and interaction database.

Types of RNA Associations	Database
mRNA-miRNA	multiMiR [16]
mRNA-lncRNA	starbase V3.0 [17]
miRNA-lncRNA	LncBase V2.0 [18]
mRNA-mRNA	STRING [19]

**Table 2:** Colon cancer-related RNAs and database.

Types of RNA	Database
mRNA	Comparative Toxicogenomics Database [20]
miRNA	miR2disease [21]
lncRNA	LncRNADisease [22]

**Table 3:** Edge Information in Heterogeneous Networks.

Type of RNA	Number of RNA	Number of Normal Samples	Number of Tumor Samples
mRNA	116591	41	443
miRNA	302	8	444
lncRNA	1526	41	443

**Table 4:** Edge Information in Heterogeneous Networks.

Type of Edge	Number of Node	Number of Edge
mRNA-mRNA	1300 (mRNA)	7408
miRNA- miRNA	81 (miRNA)	389
lncRNA-lncRNA	389 (lncRNA)	604
mRNA-miRNA	56 (mRNA) - 326(miRNA)	569
mRNA-lncRNA	33 (mRNA) - 70(lncRNA)	94
miRNA-lncRNA	99 (miRNA) - 57(lncRNA)	587

**Table 5:** Top 10 RNAs and Related Diseases.

Top 10 RNAs	Number of RNA	Related Diseases
hsa-miR-2682-5p	miRNA	Oral squamous cell carcinoma
hsa-miR-1277-3p	miRNA	/
ANGPTL1	mRNA	Lung cancer, breast cancer, colorectal cancer
SLC22A18AS	mRNA	Lung adenocarcinoma
FENDRR	lncRNA	Gastric cancer, lung cancer, hepatocellular carcinoma (HCC), gastric cancer
PHLPP2	mRNA	Diabetes, hepatic steatosis, and cancer
hsa-miR-302a-5p	miRNA	Endometrial carcinoma, glioma and breast cancer
APCDD1	mRNA	Breast cancer
MEX3A	mRNA	Glioma
hsa-miR-509-3-5p	miRNA	Gastric cancer

## Data Availability

The data are available upon request from the corresponding author.
